# Does improvement of railway network promote urban economic growth? evidence from Northeast China

**DOI:** 10.1371/journal.pone.0309133

**Published:** 2024-10-17

**Authors:** Huiying Gao, Yu Zhang, Guangquan Li

**Affiliations:** School of Geographical Sciences, Northeast Normal University, Changchun, China; AUM: American University of the Middle East, KUWAIT

## Abstract

The railway network connects different cities and regions and plays an important role in the transportation system. This paper theoretically analyzes the causal effect and mechanisms of the railway network on urban economic growth, and explores the direct effects, threshold effects, and spatial spillover effects of improved centrality in the railway network on urban economic growth in Northeast China. The results show that: (1) There is a similarity between the economic growth level and the trend of the railway network in Northeast China. From a spatial perspective, there are regional differences in the development of the railway network. (2) From the perspective of the direct effects, each unit improvement in railway network centrality leads to a 0.160 unit increase in urban economic growth in Northeast China. The degree of influence of other variables on urban economic growth, in descending order, is labor employment level, material capital investment, information development level, technological development level, human capital, and degree of openness. (3) From the perspective of the threshold effects of the railway network, the threshold values and the number of threshold levels are different for different regions. (4) There are significant spatial spillover effects in the railway network of Northeast China, and an increase in the centrality of a city’s network can have negative spatial spillover effects on the economies of surrounding areas. Based on these results, we recommend that Northeast China should focus on the construction and improvement of the railway network, strengthen the connections between regions, and promote the overall economic growth of the region while improving its international competitiveness.

## 1. Introduction

China’s economy has seen impressive growth, especially in its transportation infrastructure [1]. In 2020, China’s GDP surpassed 100 trillion yuan, with substantial progress in its transport systems, which now encompass railways, highways, aviation, river transport, and pipelines. By 2020, China had built a railway network of 146,300 km, ranking second globally, a highway network of 5,198,100 km, and a scheduled flight network of 9,426,300 km. Consequently, the role of transport infrastructure in driving economic growth has gained significant scholarly attention.

Since the 1980s, advanced transport infrastructure has been recognized as a crucial driver of China’s rapid economic growth, impacting urban spatial layout, regional economies, and economic development along transportation routes [2,3]. Initially, transportation’s role was seen in terms of improving accessibility and promoting national wealth growth. However, scholars soon began exploring the intricate relationship between transport infrastructure and economic growth, focusing on transmission effects. Empirical evidence showed that increased public investment in infrastructure precedes economic growth, attracts private investment, and spurs employment and development [4].

Early researches on how transportation development impacts economic growth initially focused on the convenience of transportation. Rosenstein-Rodan’s theory suggested that infrastructure serves as a critical foundation for economic development, though empirical evidence showed varying outcomes in developing countries. Scholars then began examining the dynamic relationship between transport infrastructure and economic growth, concentrating on transmission effects. In the 1970s, while the United States experienced a drop in public capital stock, infrastructure like roads, airports, and public transportation continued to strongly influence productivity [5]. Subsequently, more researchers explored the impact of transport infrastructure on economic growth in countries like the United States, Canada, and India [6]. These studies demonstrated that increased public investment in infrastructure preceded economic growth, attracting private investment and fostering employment, thus acting as an engine for national and regional development. Historical data analysis highlighted a strong connection between transportation volume growth and GDP growth, although this relationship doesn’t grow linearly. Once transportation reaches a certain point, its contribution to GDP growth levels off, emphasizing the need to regulate transport growth for sustainable economic development [7].

Scholars worldwide have given considerable attention to the significance of railways within transportation infrastructure research. Empirical studies in the American Midwest, Japan, and Europe have revealed the profound impact of railway construction on enterprise distribution, market expansion, urbanization, and resource allocation efficiency [8]. Research has shown that the rapid expansion of rail network strengthens city connections, fosters economic integration, and drives development in neighboring regions, promoting coordinated regional economies [9,10]. However, it’s worth noting that in some cases, expanding railway network may further benefit already industrialized areas and widen economic development disparities [11]. In China, the transportation infrastructure consists of four major components: railways, highways, civil aviation, and water transport [12,13]. Railways, in particular, handle more than 85% of both freight and passenger traffic in the country, playing a significant role in passenger flow and logistics transportation. Additionally, railway transportation has strengthened connections with other modes of transportation, establishing its dominant position within the Chinese transportation network. Therefore, this study primarily investigates the impact of railway network construction on economic growth.

Reviewing existing literature, we’ve identified the following shortcomings:

1. Previous studies mainly rely on physical indicators like railway mileage, transport investment, cargo turnover, and manpower input to measure transport infrastructure. These indicators do not fully capture the actual traffic connections between cities, particularly neglecting the importance of the relational network characteristics of transportation and the economic benefits associated with cities’ stronger links to the railway network.
2. From a methodological perspective, past research frequently uses these physical indicators to assess transport infrastructure, overlooking the relational network aspects of transportation and the economic advantages of cities being more integrated into the railway network.
3. Existing research lacks a comprehensive explanation of the micro-mechanisms through which transport infrastructure drives economic growth and often fails to systematically demonstrate various effects based on a theoretical foundation.

To address these issues, our study offers the following enhancements:

1. We take a network perspective, constructing a railway network using frequency data to understand city-to-city transportation links. We assess whether increased railway network centrality translates into real economic benefits, providing fresh empirical insights for ongoing debates.
2. Our study delves deeply into the mechanisms through which transport infrastructure drives economic growth, seeking to explain why scholars draw different conclusions regarding its impact in various regions.
3. We explore the threshold effects of railway network development on economic growth, acknowledging that its contribution can decrease after a certain point. Focusing on China’s Northeast region, we aim to pinpoint the threshold value for the railway network’s impact and propose future development policies.

Our research spans from 2006 to 2020, allowing for the continuous availability of railway data while capturing the Northeast China network’s evolution. By examining the benefits, influence mechanisms, threshold effects, and spatial impacts of railway network changes on economic development, our study aims to guide future transport policies, suggest investment directions and intensities for transportation infrastructure, and encourage coordinated economic development.

## 2. Mechanisms and models

### 2.1. The mechanism of railway network’s impact on economic growth

**Fig 1:**
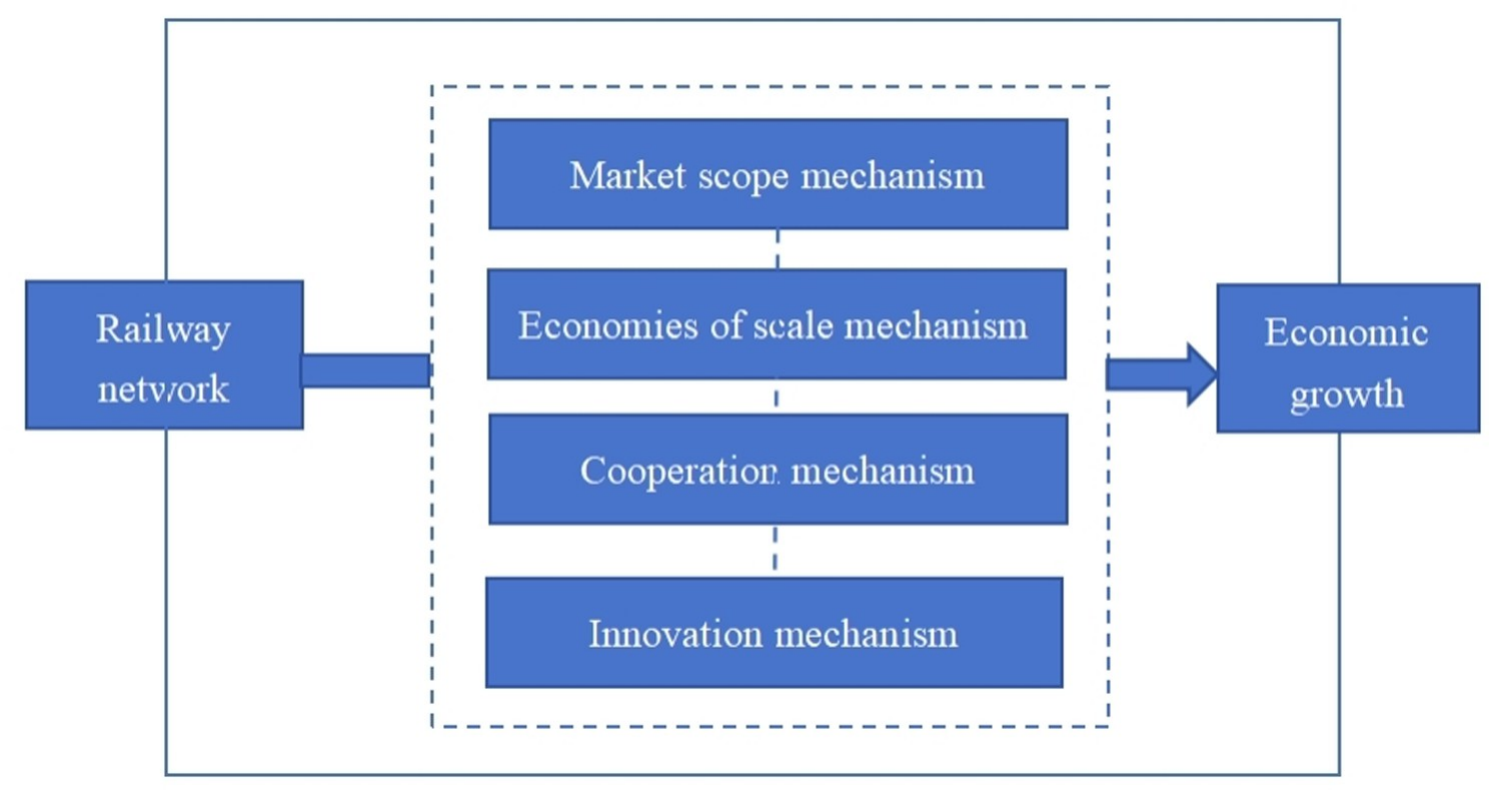
The mechanisms of railway network’s impact on economic growth.

1. Improve market allocation efficiency
  Fig 1 shows four mechanisms by which the railway network affects economic growth. The development of railway network has reduced the constraint of spatial distance on economic activities, enhanced accessibility between cities, expanded market scope, improved market allocation efficiency, reduced market transaction costs, and thus promoted economic growth.
2. Promote industrial division of labor and collaboration
  The development of railway network promotes the integration of regional functions and collaborative relationships, and cities promote the improvement of division of labor efficiency through cooperation. Different cities can achieve flexible division of labor based on node status, and achieve economic growth by integrating industrial division of labor and cooperation.
3. Promote the generation of economies of scale
  From the perspective of economies of scale, as the number of nodes in the railway network increases, the channels of factor flow between cities will become more diversified, thereby promoting the improvement of division of labor efficiency and significantly reducing the production cost of the entire network system. Promote the emergence of economies of scale and economic growth.
4. Strengthen knowledge diffusion and technology spillover

The development of railway network can promote the flow and dissemination of knowledge among enterprises in the region. The effect of collective learning is not only conducive to knowledge spillover and sharing, improving the rate of knowledge accumulation, but also enhancing the overall innovation capacity of the region, thereby promoting overall economic growth.

### 2.2. Basic hypothesis

Based on the theoretical analysis above, three basic hypotheses are considered:

#### (1) Changes in transportation infrastructure impact the economy growth

Scholars generally agree that investments in transportation infrastructure can boost GDP growth. This happens because it enhances accessibility, reduces transportation costs, improves interregional communication, and strengthens social and economic connections.

#### (2) Regional economies may experience threshold effects from the railway network, and these thresholds can differ among regions

Studies in China have shown that the relationship between railway network development and economic growth isn’t always linear. As transportation infrastructure improves, it initially aids growth, then slows down, and eventually picks up again. Different regions may have distinct threshold values for the railway network’s impact on economic growth. For instance, the effects of transportation infrastructure investment vary significantly across regions and industries [14].

#### (3) Railway network can have notable spatial spillover effects on regional economies, which should be considered to avoid overestimating the role of transportation infrastructure in regional economic growth

Research in China suggests that transportation infrastructure significantly contributes to regional economic growth through spatial spillover effects. Railways, in particular, have a substantial cross-regional impact and show increasing returns over time. The direction of these spillover effects, whether positive or negative, depends on factors like spatial scale and time. Transportation infrastructure tends to accumulate effects over time, and regions closer to transportation hubs benefit more. Additionally, short-term disruptions are believed to eventually turn into long-term driving forces [15,16]. Similar findings have also been observed in economic growth studies in regions such as Singapore and South Korea [17].

### 2.3. Construction of the models

#### 2.3.1. Direct effect model

In this section, we introduce three empirical models, including a basic model, a threshold panel model, and a spatial econometric model. We use a basic model to test Hypothesis 1. In economic growth studies, the Cobb-Douglas function is commonly used as the fundamental production function. Building on the work of other researchers, we introduce the railway network (*D*) into the growth equation as a factor affecting total factor productivity. We incorporate it into the Cobb-Douglas production function using a natural exponential function to study how changes in the railway network impact economic growth. The aggregate production function is expressed as follows:

$$Y=\left(A{\text{e}}^{\lambda \text{D}}\right)K^{a}L^{\beta }T^{\gamma }$$ (1)

In Eq (1), *Y* stands for the total output level, *K* represents capital stock, *L* denotes labor input, *T* signifies technology level, *α* represents capital output elasticity, *β* represents labor output elasticity, and *γ* represents technology output elasticity. *A* represents total factor productivity, which encompasses other factors influencing economic growth besides capital, labor, and technology.

By taking the logarithm of both sides of Eq (1), we arrive at the basic regression model, represented by Eq (2):

lnY=lnA+αlnK+βlnL+γlnT+λlnD+ε (2)

#### 2.3.2. Threshold effect model

Economic laws can be non-linear, which means that the way the railway network development impacts economic growth might have a threshold variable. To explore the dynamic and non-linear effects of railway network improvements on regional economic growth, this study employs a threshold regression model based on Hansen’s panel data threshold model. If the null hypothesis is rejected, it suggests the presence of threshold effects, and further tests can determine the specific threshold value. In the case of a significant single-variable threshold test, we also explore double-threshold and multiple-threshold models. Using a double-threshold value as an example, the model is structured as follows:

$$y_{\text{it}}=\mu_{i}+\beta_{1}^{'}x_{it}\;I\left(q_{it}\leq \gamma_{1}\right)+\beta_{2}^{'}x_{it}\;I\left(\gamma_{1}<q_{it}\leq \gamma_{2}\right)+\beta_{3}^{'}x_{it}\;I\left(q_{it}>\gamma_{2}\right)+\varepsilon_{it}$$ (3)

In Eq (3), *y*_*it*_ is the dependent variable, with *i* and *t* representing individual and time periods, respectively. *β*_*1*_*'*, *β*_*2*_*'*, and *β*_*3*_*′* are the parameters we need to estimate, while *x*_*it*_ is the explanatory variable. *ε*_*it*_ stands for the random error term, which is not correlated with the explanatory variable. *q*_*it*_ is the "threshold variable" used to split the sample, which, in this study, is the centrality of the railway network and can be one of the explanatory variables. *γ*_*1*_ and *γ*_*2*_ are the threshold values that we estimate using an indicator function ***I***(), which equals 1 if the expression in parentheses is true and 0 otherwise.

#### 2.3.3. Spatial econometric models

The previous sections delved into the influence of the railway network on economic growth using traditional econometric models. To account for the spatial aspects of the railway network, a spatial econometric model is employed to explore how changes in the railway network affect economic growth. Spatial econometric models help us understand and measure the ripple effects of individual observations in both space and time. We make use of three spatial models: the Spatial Lag Model (SLM), the Spatial Error Model (SEM), and the Spatial Durbin Model (SDM) to enhance our basic regression model. We identify the optimal model through estimation within these options. Here’s how the optimal model is expressed:

(1) Spatial Lag Model: This model links the change in the dependent variable’s spatial location with variables in different locations. It indicates that the dependent variable in a specific region depends on the dependent variable of other units. The expression is:

Y=ρWY+Xβ+ε (4)

(2) Spatial Error Model: This model primarily describes the impact of random error terms (factors not considered in the model) in other regions on the dependent variable in a given region. The expression is:

Y=Xβ+μ,μ=λWμ+ε (5)

(3) Spatial Durbin Model: It recognizes that spatial relationships are multidimensional and that spatial correlation can exist not only in the dependent variable but also in the explanatory variables. It combines aspects of the Spatial Lag Model and Spatial Error Model. The expression is:

Y=ρWY+Xβ+θWX+ε (6)

In Eqs (4)–(6), *Y* and *X* represent the dependent and explanatory variables, respectively. *ρ* is the spatial lag coefficient, reflecting the direction and extent of the influence of observations in other regions on the observation in the specified region. *β* is the explanatory variable coefficient. *μ* is the spatial error term. *λ* is the spatial error coefficient, measuring the impact of errors in other areas on the observation in the given area. *ε* is the random error term. *θ* is the spatial lag regression coefficient, representing the impact of independent variables from other regions on the dependent variable in the given region. *W* is the exogenous spatial weight matrix, showing the level of interdependence between different cities.


$$ln\;Y=\beta_{0}+\beta_{1}\text{ln}D+\beta_{2}\text{ln}C+\beta_{3}\text{ln}K+\beta_{4}\text{ln}T+\beta_{5}\text{ln}O+\beta_{6}\text{ln}U+\beta_{7}\text{ln}E+\beta_{8}\text{ln}I+\varepsilon$$ (7)

$$\begin{array}{c} ln\;Y=\beta_{0}+\beta_{1}\text{ln}D\;I\left(D\leq \gamma_{1}\right)+\beta_{2}\text{ln}D\;I\left(\gamma_{1}<D\leq \gamma_{2}\right)+\beta_{3}\text{ln}D\;I\left(D>\gamma_{2}\right)+\beta_{4}\text{ln}C+\beta_{5}\text{ln}K+\\ \beta_{6}\text{ln}T+\beta_{7}\text{ln}O+\beta_{8}\text{ln}U+\beta_{9}\text{ln}E+\beta_{10}\text{ln}I+\varepsilon \end{array}$$ (8)

$$ln\;Y=\rho W\;\ln\;Y+\beta_{0}+\beta_{1}\text{ln}D+\beta_{2}\text{ln}C+\beta_{3}\text{ln}K+\beta_{4}\text{ln}T+\beta_{5}\text{ln}O+\beta_{6}\text{ln}U+\beta_{7}\text{ln}E+\beta_{8}\text{ln}I+\varepsilon$$ (9)

$$ln\;Y=\beta_{0}+\beta_{1}\text{ln}D+\beta_{2}\text{ln}C+\beta_{3}\text{ln}K+\beta_{4}\text{ln}T+\beta_{5}\text{ln}O+\beta_{6}\text{ln}U+\beta_{7}\text{ln}E+\beta_{8}\text{ln}I+\lambda W\mu +\varepsilon$$ (10)

$$\begin{array}{c} ln\;Y=\beta_{0}+\beta_{1}\text{ln}D+\beta_{2}\text{ln}C+\beta_{3}\text{ln}K+\beta_{4}\text{ln}T+\beta_{5}\text{ln}O+\beta_{6}\text{ln}U+\beta_{7}\text{ln}E+\beta_{8}\text{ln}I+\rho W\;\text{ln}Y+\\ \lambda_{1}W\text{ln}D+\lambda_{2}W\text{ln}C+\lambda_{3}W\text{ln}K+\lambda_{4}W\text{ln}T+\lambda_{5}W\text{ln}O+\lambda_{6}W\text{ln}U+\lambda_{7}W\text{ln}E+\lambda_{8}W\text{ln}I+\varepsilon \end{array}$$ (11)

### 2.4. Data and variables

**Fig 2:**
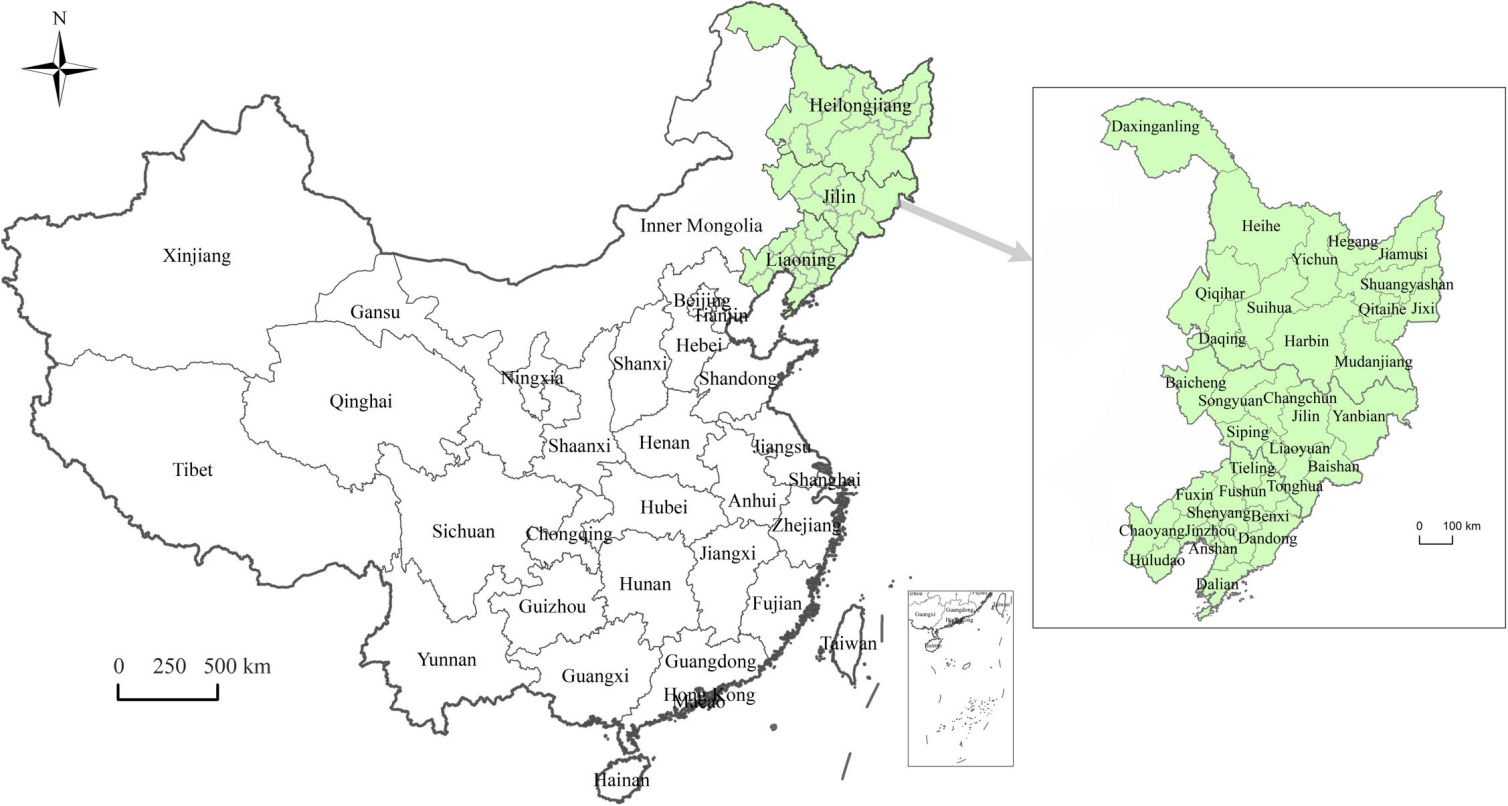
The location of Northeast China.

As of 2020, Northeast China consists of three provinces: Liaoning, Jilin, and Heilongjiang. Liaoning has 14 cities, Jilin has multiple cities and an autonomous prefecture, and Heilongjiang has 12 cities and a prefecture. We excluded Yanbian Autonomous Prefecture in Jilin and Daxinganling Prefecture in Heilongjiang due to missing data, leaving us with a total of 34 cities for this study. You can see their locations in Fig 2.

We collected data for the explanatory and control variables from various sources, such as the "Statistical Yearbook of Liaoning Province," "Statistical Yearbook of Jilin Province," "Statistical Yearbook of Heilongjiang Province," and the "China Regional Economic and Social Statistical Yearbook" from 2007 to 2021. When there were data gaps, we used linear interpolation to ensure continuity. Regarding railway network construction, we obtained schedule data from software like the "Super Timetable" and "Famous Timetable." For example, we extracted the 2006 railway schedule data from the June 21, 2006 version of the "Super Timetable" software.

The dependent variable is the level of economic growth (represented by *Y*), measured as per capita GDP in each prefecture-level city. We used a chain-weighted index to account for price fluctuations. Following the method proposed by our colleagues [18], we divided total GDP by the resident population to calculate per capita GDP and adjusted it to 2006 as the base year for comparison.

The principal variable of this study is a city’s importance in the railway network, referred to as "degree centrality" and represented as "*D*". Degree centrality measures how many connections a city has with other cities in the network. It describes a city’s position in the railway network: the higher the degree of centrality, the more central and accessible the city is within the network. Calculating this variable involves three steps:

1. We used the method developed by our colleagues [19] to collected daily train frequency data between 34 cities from 2006 to 2020 using the "Super Timetable" and "Famous Timetable" software‥ If there are multiple train stations within a city, the number of daily trains is equivalent to the non-interchange traffic between two cities. If a transfer is needed between two cities or if there’s no direct transfer station, the connection strength between those cities is set to zero.
2. Using the daily train frequency data, we created a matrix that shows the transportation connections between all 34 cities.
3. We used Ucinet software to calculate the degree of centrality of the network, which helped us analyze the role and position of each city within the railway network.

In this paper, we examine several variables, as shown in Table 1.

**Table 1:** The connotation of variables.

Variable	Code	Explanation of variables
Per capita GDP	*Y*	Per capita GDP can objectively reflect the level and degree of economic growth of a country. Therefore, the paper chooses the real per capita GDP of 34 cities after eliminating price changes to represent economic growth.
degree centrality	*D*	Degree centrality measures how many connections a city has with other cities in the network, it describes a city’s position in the railway network.
Capital input	*C*	Investment is a crucial driver of economic growth, and capital input plays a significant role. We measure it by per capita fixed asset investment in each province.
Level of human capital	*K*	Human capital influences labor and capital efficiency, impacting economic growth. We use the number of university students per 10,000 people as an indicator based on research by Huang et al. [18]
Level of technological development	*T*	Technological advancements improve input-output efficiency and drive economic growth. We typically use the number of patent applications per capita in each province to gauge technological innovation.
Degree of openness	*O*	Greater openness to the world means increased factor flow and integration into the global division of labor, enhancing city connectivity and promoting economic growth. The degree of openness is measured by the proportion of total import and export value to GDP in each province.
Level of urbanization	*U*	Urbanization boosts factor agglomeration, industrial efficiency, and economic growth. It’s typically measured as the proportion of the urban population at the end of each year to the total population in each area.
Employment level	*E*	Regional employment reflects the macroeconomic development situation directly. Stable employment brings stable labor supply, which ensures economic growth. The employment rate is the ratio of employed people to resident population.
Level of informatization development	*I*	With the increasing importance of information technology, a city’s economic growth is closely tied to its level of informatization. This is measured by the number of international internet users.

## 3.Results

### 3.1. The economic growth in Northeast China

**Fig 3:**
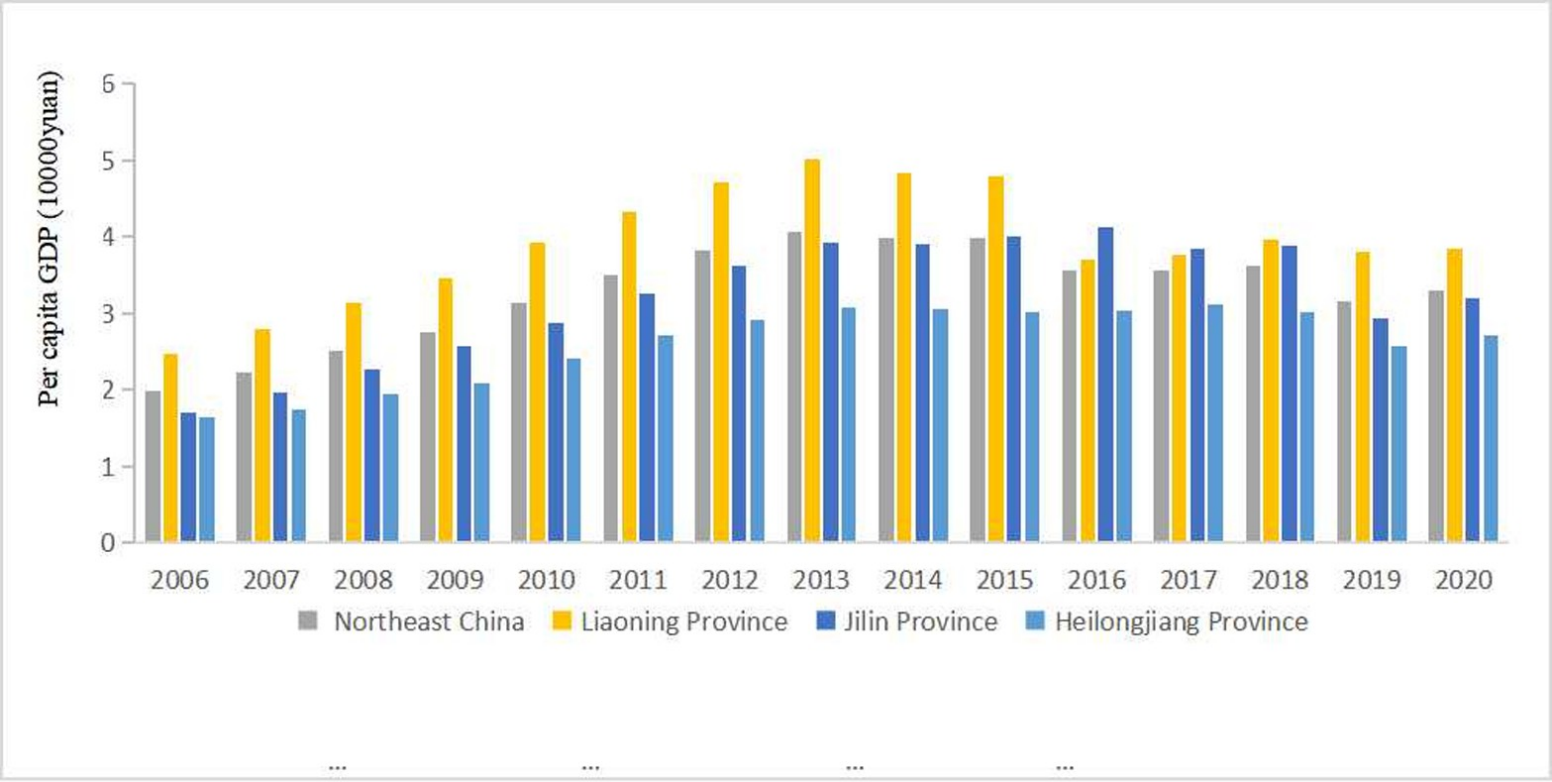
The changes of per capita GDP in Northeast China from 2006 to 2020.

From a national vantage point, the proportion of Northeast region’s total economic yield in China fell from 9.871% to 4.988% between 2006 and 2020, inferring that the region’s economic growth has lagged behind the national average. As explicated in Fig 3, the per capita GDP of Northeast China follows a pattern of initial growth, subsequent decline, and eventual stabilization. Of note, Liaoning Province’s per capita GDP consistently outstrips that of Jilin and Heilongjiang Provinces. Analyzing the development trend of per capita GDP in Northeast China, it is divided into two phases. From 2006 to 2013, the per capita GDP of Northeast China demonstrated an annual increase, indicating rapid economic evolution. This surge was largely attributable to China’s policy of resuscitating its traditional industrial heartlands in Northeast China coupled with the beneficial nationwide economic dynamism. Both governmental investment and policy were the linchpins of Northeast China’s economic expansion during this period. As we transition to 2014–2020, the second phase signifies a decrease and subsequent plateau in the per capita GDP of Northeast China with the GDP growth rate losing its previous vigour. Inter-provincial GDP discrepancies began to diminish during this phase. This period also witnessed a significant deceleration of global economic growth, ushering China’s economy into a new norm. However, the Northeast region, a symbolic old industrial bedrock, struggled to dovetail into these new economic dynamics, plagued by endemic issues such as resource exhaustion, rigid planning systems, and an illogical economic structure. The region struggled to acclimate to the new normal, exacerbated by a severe industrial recession and the emergence of the "New Northeast" phenomenon.

### 3.2. The development characteristics of the railway network in Northeast China

**Fig 4:**
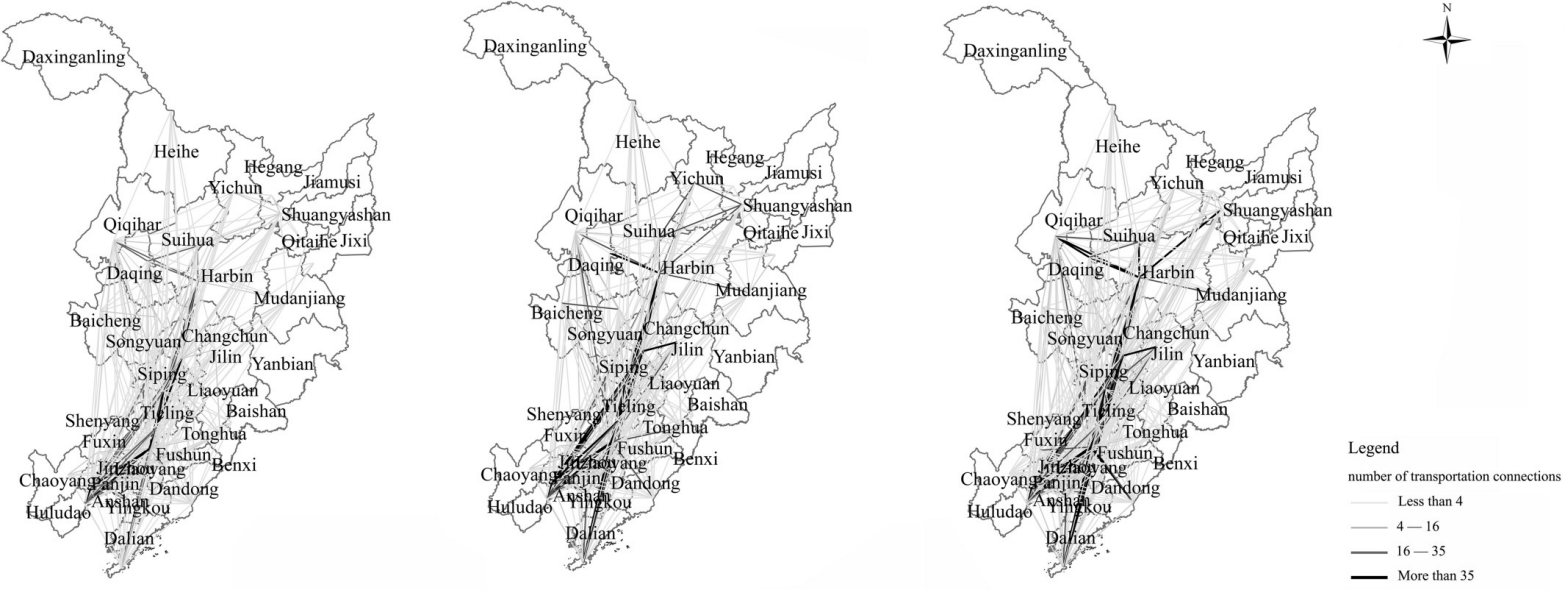
The number of connections in the railway network in 2006, 2013, and 2020.

Utilizing train schedule data, we can quantify the transportation links between cities, as illustrated in Fig 4, revealing that the trend for these links in Northeast China has generally increased before stabilizing, an evolution similar to the economic growth pattern of the region. The average number of intercity connections expanded from 3.701 in 2006 to 6.357 in 2020, demonstrating enhanced connectivity across the railway network in Northeast China and suggesting that a comprehensive railway network ensuring basic connectivity has been established. A province-wise analysis shows that Liaoning Province boasts the strongest intra-provincial connectivity, followed by Jilin and Heilongjiang Provinces, respectively. Liaoning, as Northeast China’s gateway to development with advanced economic growth and well-facilitated infrastructure such as railways, generates stronger intra-provincial connections. Jilin Province, despite being the region’s geometrical transportation hub, mainly sees concentrated interactions between major cities while smaller cities lack close ties and cross-provincial cooperation. Heilongjiang Province, given its high latitude, low population density, and harsh climate, faces challenges in building transportation facilities, which weaken intercity, and intra- and inter-provincial ties. In conclusion, despite a relatively advanced transportation system in Northeast China facilitating intercity throughput, a mature mechanism for promoting inter-provincial development remains absent in the region leading to weak economic activities and inter-regional collaborations among regional cities.

**Fig 5:**
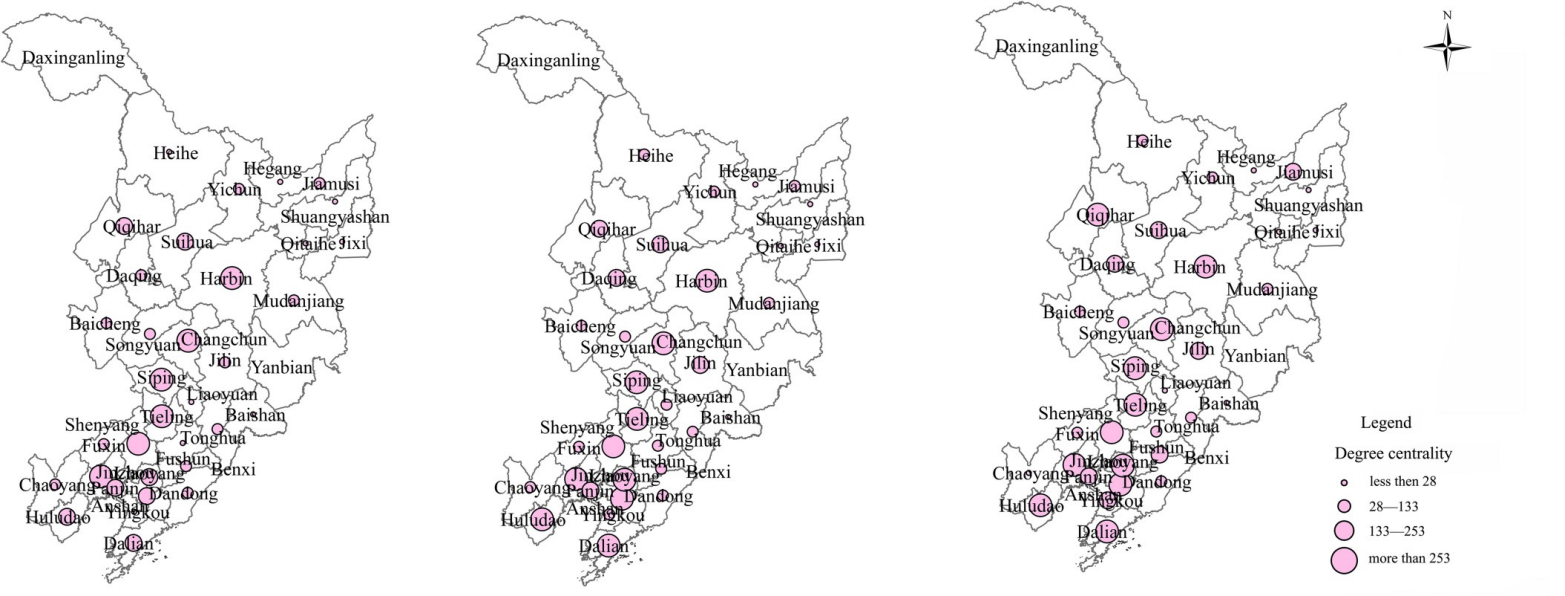
Degree of centrality of railway network in cities of Northeast China from 2006 to 2020.

This study employs Ucinet software to quantify the degree of centrality, thereby examining each city node’s role and position within the railway network. A higher degree of centrality corresponds to greater integration into the network and suggests a more developed railway network within the region. Fig 5 presents these results. The degree of centrality of the railway network across prefecture-level cities was calculated for the years 2006, 2013, and 2020, generating mean values of 56.026, 58.977, and 58.725 respectively. These values reflect a consistent upward trend over two decades, indicative of an improved railway network infrastructure. This ongoing improvement contributes to the enhancement of the network’s overall connectivity and radiating capacity. Considering spatial aspects, a clear regional disparity can be observed in the development of the railway network in Northeast China. Cities such as Shenyang, Changchun, Harbin, and Siping consistently rank high in terms of centrality, establishing their key position within Northeast China’s railway network and their crucial role as central cities. These cities demonstrate advanced levels of economic prosperity and infrastructure development and are located in resource-rich areas, thus enabling efficient transportation links with other cities. These regions are characterized by a high concentration of railway network associations, heavy economic activity, and considerable cross-regional collaboration and communication among regional cities. Conversely, prefecture-level cities such as Qitaihe and Shuangyashan typically rank low in terms of degree of centrality, indicating their marginal position within the network. Their geographical locations are less than optimal and they generally exhibit limited connectivity with other prefecture-level cities, making their transportation prominence relatively underwhelming.

### 3.3. Empirical analysis of direct effects

The first step is to perform descriptive statistics on the variables provided in Table 2, including mean, standard deviation, minimum, maximum, and observed values, in order to better understand the data characteristics of these variables. Subsequently, a correlation test between the study variables was carried out, revealing a linear correlation coefficient below 0.8 for most variables. This suggests that there is no serious multicollinearity between the variables. Finally, in order to prevent spurious regression results caused by non-stationary data, we conducted unit root tests on the panel data. The Augmented Dickey-Fuller (ADF) test and Levin, Lin & Chu (LLC) test were administered. The rejected null hypothesis of unit roots’ existence implies that the panel data used in this study are stationary.

**Table 2:** Descriptive statistics of data.

Variable	Mean	Standard Deviation	Minimum	Maximum	Observations	ADF Test Value	p-value	LLC Value	p-value
ln*Y*	10.433	0.596	8.851	11.904	510	130.3297	0.000	-9.494	0.004
ln*C*	3.822	1.076	1.199	6.518	510	202.823	0.000	-6.652	0.003
ln*K*	6.330	1.757	0.314	11.375	510	101.817	0.005	-7.037	-0.028
ln*T*	3.922	0.506	3.188	13.236	510	241.719	0.000	-13.863	-0.000
ln*O*	3.106	0.380	1.809	4.129	510	106.962	0.001	-9.264	-0.001
ln*U*	4.384	1.153	0.836	6.537	510	115.406	0.000	-10.071	-0.000
ln*E*	2.504	0.696	0.111	3.726	510	121.104	0.000	-11.165	-0.000
ln*I*	2.848	0.990	0.476	5.350	510	167.546	0.000	-12.267	-0.000
ln*D*	4.728	1.288	1.386	7.260	510	208.982	0.004	-8.683	-0.002

The railway network’s impact on economic growth was first analyzed using an ordinary least squares (OLS) regression. Before considering any other control variables, a significantly positive regression coefficient was obtained, passing the 1% significance test. This result highlights the positive impact of improving the centrality of the railway network on economic growth. The inclusion of relevant control variables made the regression coefficient more sensible and improved the model’s goodness of fit, suggesting the suitability of the selected control variables and the proper establishment of the model.

This study also conducted tests on random and fixed effects, considering individual city-specific differences. Both tests resulted in a statistic value and p-value of 139.190 and 0.000, respectively for the F-test, and 31.480 and 0.000 for the Hausman test, each passing the 1% significance threshold. This result rejects the null hypothesis of the nonexistence of fixed effects and justifies the use of a panel fixed effects model for regression. The fixed effects model focuses more on the differences between samples and can effectively measure the contribution of railway network to economic growth. Table 3 presents the coefficient estimation results, which demonstrate a slight increase in significance compared to the OLS regression. This suggests that the OLS regression may have underestimated the significance of the explanatory and control variables. The main focus would henceforth be on the regression results based on the fixed effect model.

**Table 3:** Estimated results of OLS model and fixed effects model.

Variable	OLS coefficients	t-values	p-values	fixed effects coefficients	t-values	p-values
ln*C*	0.096	3.540	0.000	0.224	6.811	0.000
ln*K*	-0.015	1.180	0.239	0.045	2.980	0.003
ln*T*	-0.086	-2.701	0.007	0.050	-1.560	0.001
ln*O*	0.739	14.351	0.000	0.036	5.243	0.000
ln*U*	0.040	2.172	0.031	-0.197	3.251	0.120
ln*E*	-0.009	-0.360	0.722	0.277	7.010	0.000
ln*I*	0.170	6.191	0.000	0.109	10.042	0.000
ln*D*	0.063	4.135	0.000	0.160	5.497	0.000
*R* ^*2*^ *F*	0.635 111.760			0.704 139.190		

After conducting fixed effect model estimations in Northeast China, the results show all variables, excluding the urbanization indicator, surpassed the significance test at a 1% level. The model fitting is deemed successful, illustrated by the R^2^ value of 0.704. The model fitting also implies negligible residual sequence correlation, given by the Durbin-Watson value of 1.963. Focusing on the model’s regression coefficients, the elasticity coefficient of the core explanatory variable, namely the railway network, is 0.160. This implies that with each unit increase in the centrality of the railway network, per capita GDP will grow by 0.160 units. Consequently, the significance of the centrality of the railway network in boosting economic growth is established. This supports the first hypothesis of the study, suggesting potential economic changes due to changes in transportation infrastructure under similar conditions.

All controlled variables excluding the urbanization indicator have surpassed the significance test, demonstrating a significant positive impact on Northeast China’s economic growth. The degree of influence in descending order is the level of labor employment, the investment of materials capital, the level of information development, the level of technological development, the human capital, and the degree of openness. The driving force behind Northeast China’s economic growth primarily appears to be capital stock and labor input. This exhibits that traditional factors significantly affect economic growth. Additionally, the level of information and scientific research is essential for regional economic development, indicating that informatization and technology have a strong positive impact on economic growth. Nevertheless, the impact of the levels of human capital and openness on economic growth is relatively weak, aligning with Northeast China’s real-life circumstances such as insufficient openness, ineffective external communication, talent drain, and overall weak scientific and technological strength. The coefficient of urbanization on economic growth was negative and did not surpass the significance test. Ideally, a level of higher urbanization implies a more developed tertiary industry and urban economy. Unfortunately, Northeast China contradicts this assumption, emphasizing that urbanization does not necessarily promote economic growth, particularly when based on state-owned heavy industry with substandard quality and incomplete labor division.

### 3.4. Empirical analysis of threshold effects

Employing a fixed effect model, we found that the railway network’s development significantly impacts economic growth. However, it does not mean that the higher the centrality of the railway network, the more it can promote economic growth. The expression function may change due to a certain threshold, thus exhibiting non-linear characteristics. At present, we propose the potential existence of threshold effects between transportation infrastructure and economic growth, due to the construction and enhancement of the railway network. The panel threshold model was proposed by Hansen in 1999 and is an application and extension of traditional threshold regression models on panel data. It is a model used to handle nonlinear relationships, especially when dealing with data with interaction and asymmetric effects, which has significant advantages. Hence, guided by Hansen’s threshold model, we designated the threshold variable as the centrality of the railway network. We utilized the bootstrap method to simulate the likelihood ratio (LR) statistic and its critical value, further investigating the threshold effects. Table 3 shows the test results.

According to the test results in Table 4, the LR statistics for single and double thresholds have passed the significance test at a level of 1%, indicating that the threshold value is valid at a significance level of 1%. However, the test statistic for triple threshold did not pass the significance test, indicating rejection of the null hypothesis and thus rejecting the existence of three thresholds. Therefore, it was determined that there are two threshold values for the centrality of railway network nodes. In addition, we conducted a heterogeneity test by province, which showed that the impact of railway network on economic growth in Liaoning Province exhibits double threshold characteristics, while Jilin Province exhibits single threshold characteristics, and Heilongjiang Province exhibits double threshold characteristics.

**Table 4:** Results of panel threshold model test.

Region	Threshold count	F-statistics	p-values	Result
Northeast China	Single threshold	19.8	0.000	Accept
	Dual threshold	18.5	0.000	Accept
	Triple threshold	9.28	1.000	Reject
Liaoning province	Single threshold	13.95	0.007	Accept
	Dual threshold	19.38	0.000	Accept
	Triple threshold	8.28	0.667	Reject
Jilin province	Single threshold	50.45	0.000	Accept
	Dual threshold	7.65	0.333	Reject
	Triple threshold	26.98	0.333	Reject
Heilongjiang province	Single threshold	15.66	0.000	Accept
	Dual threshold	17.01	0.000	Accept
	Triple threshold	5.59	0.667	Reject

Note: The values in parentheses indicate P-values.

Based on the model’s estimation results, the estimated outcomes of the control variables align with the regression results of fixed effects from the first section. This consistency suggests that the chosen control variables are robust and reasonable; further analysis won’t be conducted. We will primarily analyze the core explanatory variable. Table 5 demonstrates that given other variables remain constant, the railway network centrality’s influence on Northeast China’s economic growth can be segregated into three intervals, considering the double threshold values of 1.610 and 4.564. These have passed the 1% significance test level, indicating a steadfast, positive effect on economic growth by enhancing the railway network. When the degree centrality of the railway network falls within the three ranges of less than 1.610, 1.610 to 4.564, and exceeding 4.564, it leads to a per capita GDP growth of 0.392%, 0.179%, and 0.209%, respectively.This suggests that the early railway network development spurs significant economic growth. Nevertheless, as the railway network evolves, its impact on regional economic advancement gradually diminishes. It is not until breaching the second threshold that its economic growth effects re-inflate slightly. Hence, blindly seeking excessive investments in transportation infrastructure shouldn’t be the primary focus of cities. Instead, they should pursue railway network scales in harmony with economic development.

**Table 5:** Estimated results of the panel threshold regression model.

Threshold ranges for *D*	Northeast China	Coefficient	Liaoning Province	Coefficient	Jilin Province	Coefficient	Heilongjiang Province	coefficient
Interval 1 Interval 2 Interval 3	<1.610 1.610–4.564 >4.564	0.392*** 0.179*** 0.209*	<3.332 3.332–6.687 >6.687	0.235*** 0.185** 0.147**	<4.956 >4.956	0.319** 0.194*	<1.609 1.609–4.357 >4.357	0.380*** 0.150** 0.205*

Note: *, **, and ***denote significance at the 10%, 5%, and 1% levels, respectively.

In the provincial test results, it can be found that there are differences in the threshold effect of railway network enhancement on economic growth among different provinces. The dual thresholds for the railway network in Liaoning Province are 3.332 and 6.687, respectively, while the single threshold for Jilin Province is 4.956. After the centrality of the railway network in both provinces exceeds the threshold, the impact on economic growth weakens. The possible reason is that when the city’s embedding degree in the railway network is low, the industrial development and market competitiveness are relatively weak, and it is more necessary to make up for its shortcomings through the railway network. The level of economic growth is more sensitive to the development of the railway network, and the degree of benefit is greater. When a city is highly embedded in the railway network, it often has a larger population size and resources, and has established a certain advantageous position in market competition. Relatively speaking, the impact coefficient of the railway network on economic growth decreases. The dual thresholds for Heilongjiang Province are 1.609 and 4.357, and the centrality of the railway network shows a characteristic of first decreasing and then increasing after crossing the threshold. The possible reason is that some small cities in Heilongjiang Province are located on the edge, and the early construction of railway network had a driving effect on economic growth, but there was a phenomenon of production factor outflow. Therefore, after crossing the first threshold, the degree of impact decreased. With the continuous development of railway network, the driving force of economic growth further increased, until after crossing the second threshold, production factors and resources were further allocated, and the impact on economic growth increased again. Among the three provinces, Heilongjiang Province has the lowest threshold, indicating that compared with Liaoning and Jilin provinces, the infrastructure development in Heilongjiang Province is lagging behind, with a smaller scale and still in the primary development stage. A slight improvement in the railway network can trigger economic transformation. Therefore, we confirm hypothesis 2, which states that the threshold effect of railway network on economic growth varies in different regions.

### 3.5. Empirical analysis of spatial spillover effects

Based on the fixed effects model mentioned above, spatial econometric models were used to examine the spatial effects of changes in railway network on economic growth by introducing a weight matrix to consider spatial correlations. The spatial econometric model mainly includes three types: spatial lag model, spatial error model, and spatial durbin model. The specific model selection was determined by estimating the results. Firstly, based on the fixed effects model mentioned above, LM, Wald, and LR tests were conducted sequentially. The test results are shown in Table 6, which rejected the null hypothesis and passed the significance test at the 1% level. Therefore, the optimal model is the SDM model, and its parameter results will be discussed. In addition, the SDM model can consider the spatial effects of railway network, that is, the increase in centrality of a regional railway network not only affects local economic growth, but also has an impact on the economic growth of surrounding areas.

**Table 6:** The results of model validation.

Variable	Statistic	p-values
LM-Lag	107.132	0
R-LM-Lag	94.057	0
LM-Err	312.011	0
R-LM-Err	192.425	0
Wald test for spatial lag	112.108	0
LR test spatial lag	99.034	0
Wald test for spatial error	104.092	0
LR test spatial error	213.107	0

To affirm the robustness of the research, Table 7 provides estimates from the spatial durbin model using diverse weight matrices. The spatial lag coefficientρ And spatial error coefficient λ are significantly positive, indicating significant spatial correlation between regional economic growth and factors not included in the model. On the other hand, the spatial effects under these three weights pass the significance test at the 1% level, reaffirming the spatial spillover effects of the railway network on economic growth. This lends weight to Xu’s conclusion that the spillover effects from economic links eclipse those resulting from simple adjacency and geographic distance.

**Table 7:** Estimated results of the spatial durbin model.

Spatial effects	Based on Spatial Adjacency Matrix	Based on Geographic Distance Matrix	Based on Transportation Connectivity Matrix
W*ln*C*	-0.096 *** (0.000)	-0.354*** (0.000)	-0.281*** (0.000)
W*ln*K*	-0.015*** (0.000)	0.118 (0.239)	-0.197*** (0.003)
W*ln*T*	0.086(0.100)	-0.270 (0.107)	0.156 (0.120)
W*ln*O*	0.239***(0.000)	0.143 *** (0.000)	0.124*** (0.000)
W*ln*U*	0.040*(0.090)	0.217 ** (0.031)	0.325*** (0.001)
W*ln*E*	-0.009**(0.042)	-0.361 (0.722)	-0.170*** (0.000)
W*ln*I*	0.170***(0.000)	0.192 *** (0.000)	0.104 *** (0.000)
W*ln*D*	-0.063*** (0.000)	-0.113** (0.020)	-0.149 ***(0.000)
ρ	0.294***(0.000)	0.146***(0.000)	0.113***(0.000)
λ	0.011***(0.000)	0.009***(0.000)	0.023***(0.000)
R^2^	0.764	0.7177	0.7701

Note: P-values are shown in parentheses,: *, **, and ***denote significance at the 10%, 5%, and 1% levels, respectively.

In examining regression coefficients of individual variables, we find alignment with previous studies in terms of sign, albeit with varying magnitudes. This points to a stable model setup. Our analysis focuses primarily on the spatial spillover effects of each variable. Improved centrality of the railway network reveals negative spatial spillover effects on other cities financially. An interpretation could be that increased centrality inhibits the economic growth of adjacent areas, probably because a region with superior transportation attracts the inflow of production factors from neighboring regions. Correspondingly, an increase in local capital stock and labor force, indicated by spatial lags of fixed capital, human capital level, and employment rate, imposes similar limitations. This can be attributed to most factors tilting towards developed city centers in order to achieve higher returns, which is detrimental to underdeveloped neighboring cities such as human resources, labor, and capital. The strength of polarization surpasses diffusion, hence the overall negative spillover. Although the spatial lag of technological input has a positive factor, it lacks significance, implying a lack of clear spillover effects at the technology level. This is likely due to the persistently low levels of technological development in Northeast China. On the contrary, the spatial spillover effect of information and openness is positive, and a region may stimulate economic progress in nearby cities. Therefore, government policies in the Northeast region should recognize the spatial interdependence between cities and the interaction of factors such as capital, labor, and economy. It is crucial to carefully adapt transportation policies to the environment in order to mitigate negative spillover effects.

### 3.6. Endogeneity testing

The static panel fixed effects model was used for regression in the previous text. Considering the possible endogeneity in the model, the system GMM was used to test the relationship between railway network and economic growth, in order to reflect the dynamic lag effect of economic growth. Therefore, a lagged period of economic growth level is added as the explanatory variable, and a dynamic panel data regression model is established. The system generalized moment estimation method is used to estimate the parameters and further analyze the robustness of the model. Firstly, the autocorrelation of the disturbance term in the established model was tested. From Table 8, it can be seen that the test values of the traffic network models AR (1) and AR (2) indicate the existence of first-order autocorrelation.

**Table 8:** Estimated results of the system GMM.

variable	Based on Spatial Adjacency Matrix	Based on Geographic Distance Matrix	Based on Transportation Connectivity Matrix
L.ln*Y*	0.013***(0.000)	0.022***(0.000)	0.018***(0.000)
ln*C*	-0.089*** (0.000)	-0.078*** (0.000)	-0.091*** (0.000)
ln*K*	-0.013*** (0.000)	0.104 (0.939)	-0.118*** (0.009)
ln*T*	0.072(0.325)	0.270 (0.602)	0.212(0.103)
ln*O*	0.121***(0.000)	0.109 *** (0.000)	0.172*** (0.000)
ln*U*	0.036**(0.030)	0.118 ** (0.027)	0.019*** (0.000)
ln*E*	-0.014**(0.030)	-0.251 (0.526)	-0.470*** (0.000)
ln*I*	0.108***(0.000)	0.119 *** (0.000)	0.082 *** (0.000)
ln*D*	-0.054*** (0.000)	-0.135** (0.020)	-0.123 ***(0.000)
AR(1)	0.004***(0.000)	0.006***(0.000)	0.003***(0.000)
AR(2)	0.710***(0.000)	0.739***(0.000)	0.823***(0.000)
Sargan	1	1	1

Note: P-values are shown in parentheses,: *, **, and ***denote significance at the 10%, 5%, and 1% levels, respectively.

From the Sargan test, the results show that the corresponding p-value is 1, which cannot reject the null hypothesis of the validity of the instrumental variable. Therefore, the setting of the system GMM estimation model is reasonable, and using the lagged term of the explained quantity as an instrumental variable is also effective. By comparing the coefficient symbols and significance levels estimated by the system GMM with the fixed effects model in Table 8, it was found that the direction of the coefficient symbols is consistent, with only slight differences in parameter values. This once again indicates that the empirical results of the impact of railway network on economic growth are robust and reliable.

## 4. Discussion

Extensive literature presents a variety of models employed by scholars to empirically investigate the effect of transportation infrastructure on economic growth. These models include, for example, neoclassical growth models, dynamic structure models, logarithmic production function models, public capital models, vector autoregressive models, cost function models, and spatiotemporal difference models [5,13,15,20–22]. Both time-series data and panel data served as the data basis for these studies, providing a wealth of information covering developed nations like Europe, the United States, and Japan, as well as developing countries such as Africa and China [8,12,23]. However, variations in research methodologies, types of data, and the economic development of examined regions can result in differing findings, depending on the scope of research and area of focus. Key debates focus on the positive or negative impact of transportation infrastructure on economic growth and spillover effects.

Our research builds on these previous studies. Leveraging fixed effect models, panel threshold models, and spatial spillover effects models, we aim to empirically analyze the effect of transportation infrastructure on Northeast China’s economic growth. These analyses provided several key insights. Firstly, a general upward trend indicates the quality of the railway network has improved over two decades, with improved connectivity and radiative capacities. A unit increase in railway network centrality resulted in a 0.160 unit surge in economic growth, showing a positive impact. Secondly, the economic growth displayed threshold effects resulting from the enhancement of the railway network. The early stages of railway network development had a dramatic and rapid positive effect on economic growth. But as the network expanded, the extent of this impact diminished before slightly increasing after breaking a second threshold. Compared to core urban economies, non-core urban economic growth benefited significantly more from transportation infrastructure. Lastly, the increasing centrality of the railway network in Northeast China resulted in negative spatial spillover effects on other cities. Importantly, the spatial spillover effects extend beyond mere geographical adjacency.

This study’s contribution lies in four critical aspects. Firstly, it moves past macroeconomic discussions of the relationship between transportation infrastructure and economic growth to systematically analyze direct effects, threshold effects, and spatial spillover effects resulting from railway network enhancement. Secondly, the research introduces the centrality of the railway network as a factor impacting economic growth, extending empirical research on railways. Thirdly, the study verifies current theories and identifies the threshold effect and negative spatial spillover effect of railway network on economic growth. Additionally, it finds that Northeast China is still in a polarized stage.

The railway network, as an important component of infrastructure, has a profound impact on the economic growth of various countries. The railway system in the United States effectively promotes transportation, trade, and personnel mobility between cities, while Europe greatly enhances economic integration and regional competitiveness through its transnational high-speed railway network. In recent years, India has also been promoting the modernization and expansion of its railway network, including the introduction of high-speed railway technology and the improvement of intercity transportation. By drawing on the development experience of other countries, it can be found that the railway network in Northeast China has to some extent promoted regional economic growth, but due to low investment efficiency and utilization rate, the direct contribution and indirect impact are relatively limited. In addition, as an important old industrial base in China, the Northeast region has faced the challenge of economic transformation in recent years. Improving transportation infrastructure, especially the modernization and efficiency of railway network, is of great significance for promoting the recovery and long-term growth of the region’s economy, and enhancing its attractiveness to enterprises and talents.

Based on our findings, we suggest several policy adjustments. Firstly, since railway network improvement had a positive impact on economic growth, investment in the railway network should increase. Despite Northeast China’s railway network density being high within China, it falls short compared to international metropolitan areas; thus, railway construction in Northeast China’s provinces should be strengthened and urban transport integration strengthened. Secondly, from the perspective of the threshold effect, the impact saturation of transportation infrastructure on economic development relies on matched growth. Thirdly, from the perspective of the spatial spillover effects, long-term support is needed for transportation infrastructure to facilitate the effective linkage between central and surrounding cities, and to expand urban economic agglomeration. Fourthly, Northeast China needs to transition towards a more innovation-driven economic growth model, boosting human capital, technological capabilities, and research result transformations. The convenience of the railway network can support the sharing of production factors and the creation of this new economic growth model.

Therefore, when planning and implementing railway network improvements, the government needs to adopt a comprehensive strategy, including continued investment in railway infrastructure, integration of urban transportation systems, introduction of new technologies, and consideration of regional realities, in order to achieve the maximum contribution of railway network improvements to economic growth. At the same time, China has rich experience and technology in railway construction, which provides technical support for implementing related projects in the Northeast region. However, there are challenges in implementing the above policies. The economic development in Northeast China is relatively lagging behind, and fiscal revenue is limited. How to raise sufficient funds for railway facility investment, coordinate the interests of all parties in the urban transportation system, ensure the smooth progress of projects and generate long-term benefits is a potential challenge. With the development of high-speed rail and high-speed train technology [24–26], considering the dynamic nature of transportation infrastructure development and its impact on economic growth, the author attempts to explore the long-term impact of high-speed railway projects on regional economic integration, or to study the role of emerging technologies such as autonomous vehicle in shaping urban economic dynamics as a future research direction.

## Supporting information

S1 Table Basic statistics of railway connections. (XLSX)

S2 Table Basic statistics of economic growth model. (XLSX)
